# Supplementary material for: Causal Relationship between Aging and Anorexia Nervosa: A White-Matter-Microstructure-Mediated Mendelian Randomization Analysis
Source: Biomedicines. 2024 Aug 16;12(8):1874. doi: 10.3390/biomedicines12081874 (PMC11351342; doi:10.3390/biomedicines12081874)
Supplement: Supplementary file 1 [file biomedicines-12-01874-s001.zip › Supplementary Figure/Figure S4/Singe SNP plot of MR analysis for TL to ubm-b-2058.pdf]

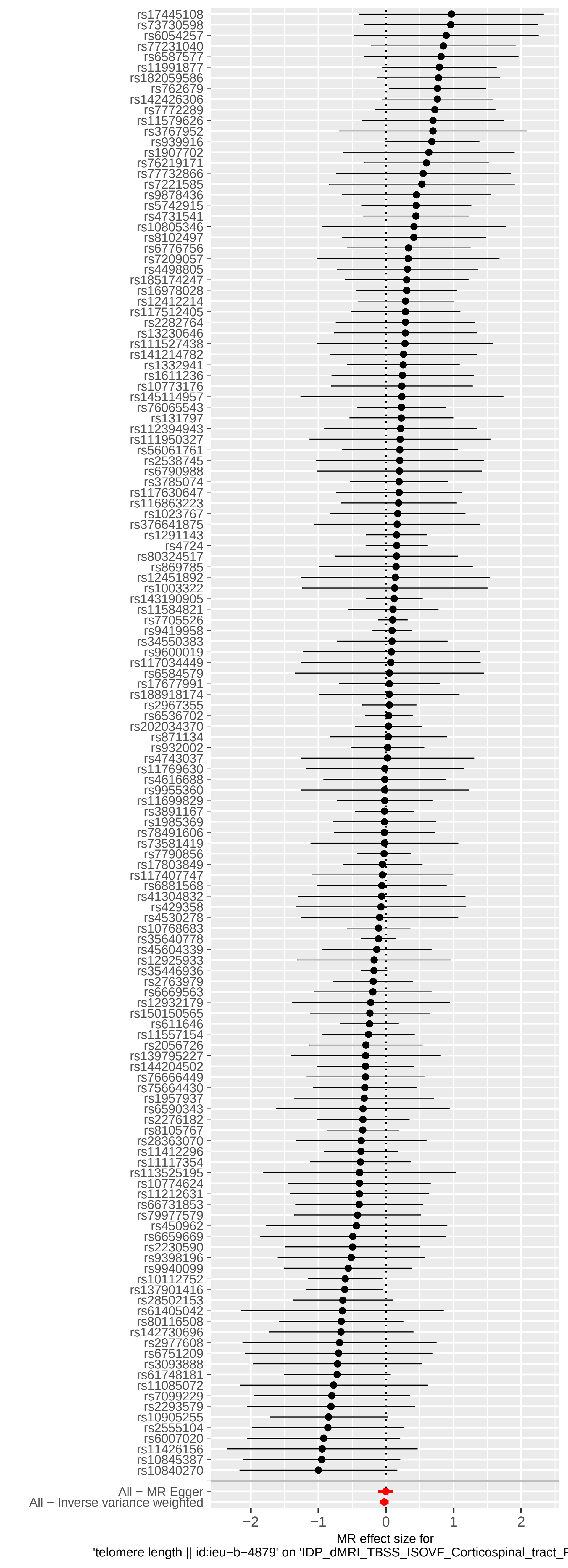

rs17445108
rs73730598
rs6054257
rs77231040
rs6587577
rs11991877
rs182059586
rs762679
rs142426306
rs7772289
rs11579626
rs3767952
rs939916
rs1907702
rs76219171
rs77732866
rs7221585
rs9878436
rs5742915
rs4731541
rs10805346
rs8102497
rs6776756
rs7209057
rs4498805
rs185174247
rs16978028
rs12412214
rs117512405
rs2282764
rs13230646
rs111527438
rs141214782
rs1332941
rs1611236
rs10773176
rs145114957
rs76065543
rs131797
rs112394943
rs111950327
rs56061761
rs2538745
rs6790988
rs3785074
rs117630647
rs116863223
rs1023767
rs376641875
rs1291143
rs4724
rs80324517
rs869785
rs12451892
rs1003322
rs143190905
rs11584821
rs7705526
rs9419958
rs34550383
rs9600019
rs117034449
rs6584579
rs17677991
rs188918174
rs2967355
rs6536702
rs202034370
rs871134
rs932002
rs4743037
rs11769630
rs4616688
rs9955360
rs11699829
rs3891167
rs1985369
rs78491606
rs73581419
rs7790856
rs17803849
rs117407747
rs6881568
rs41304832
rs429358
rs4530278
rs10768683
rs35640778
rs45604339
rs12925933
rs35446936
rs2763979
rs6669563
rs12932179
rs150150565
rs611646
rs11557154
rs2056726
rs139795227
rs144204502
rs76666449
rs75664430
rs1957937
rs6590343
rs2276182
rs8105767
rs28363070
rs11412296
rs11117354
rs113525195
rs10774624
rs11212631
rs66731853
rs79977579
rs450962
rs6659669
rs2230590
rs9398196
rs9940099
rs10112752
rs137901416
rs28502153
rs61405042
rs80116508
rs142730696
rs2977608
rs6751209
rs3093888
rs61748181
rs11085072
rs7099229
rs2293579
rs10905255
rs2555104
rs6007020
rs11426156
rs10845387
rs10840270
All − MR Egger
All − Inverse variance weighted
−2
−1
0
1
2
MR effect size for
'telomere length || id:ieu−b−4879' on 'IDP_dMRI_TBSS_ISOVF_Corticospinal_tract_R
